# Efficacy and Physicochemical Evaluation of an Optimized Semisolid Formulation of Povidone Iodine Proposed by Extreme Vertices Statistical Design; a Practical Approach

**Published:** 2015

**Authors:** Farzaneh Lotfipour, Hadi Valizadeh, Shahin Shademan, Farnaz Monajjemzadeh

**Affiliations:** a *Department of Pharmaceutical and Food Control, Faculty of Pharmacy, Tabriz University of Medical Sciences, Tabriz, Iran.*; b * Drug Applied Research Center, Tabriz University of Medical Sciences, Tabriz, Iran.*; c *Department of Pharmaceutics, Faculty of Pharmacy, Tabriz University of Medical Sciences, Tabriz, Iran. *; d *Research Center for Pharmaceutical Nanotechnology, Tabriz University of Medical Sciences, Tabriz, Iran. *; e *Student Research Committee, Tabriz University of Medical Sciences, Tabriz, Iran.*

**Keywords:** Pre-Formulation, Statistical design, Transdermal drug delivery, Povidone iodine

## Abstract

One of the most significant issues in pharmaceutical industries, prior to commercialization of a pharmaceutical preparation is the "preformulation" stage. However, far too attention has been paid to verification of the software assisted statistical designs in preformulation studies. The main aim of this study was to report a step by step preformulation approach for a semisolid preparation based on a statistical mixture design and to verify the predictions made by the software with an *in-vitro* efficacy bioassay test.

Extreme vertices mixture design (4 factors, 4 levels) was applied for preformulation of a semisolid Povidone Iodine preparation as Water removable ointment using different PolyEthylenGlycoles. Software Assisted (Minitab) analysis was then performed using four practically assessed response values including; Available iodine, viscosity (N index and yield value) and water absorption capacity. Subsequently mixture analysis was performed and finally, an optimized formulation was proposed. The efficacy of this formulation was bio-assayed using microbial tests *in-vitro* and MIC values were calculated for Escherichia coli, pseudomonaaeruginosa, staphylococcus aureus and Candida albicans.

Results indicated the acceptable conformity of the measured responses. Thus, it can be concluded that the proposed design had an adequate power to predict the responses in practice. Stability studies, proved no significant change during the one year study for the optimized formulation. Efficacy was eligible on all tested species and in the case of staphylococcus aureus; the prepared semisolid formulation was even more effective.

## Introduction

Statistical Mixture design approach allows a formulator scientist to find the effective components of a formulation mixture and propose a model according to practically measured response values which may provide a good prediction for the preformulation purposes (1). 

Mixture designs can be applied for a type of mixtures in which the proportions of the components (factors) are more important compared to their magnitude (2). The design finally leads to response optimization. Mixture design can be subdivided into simplex centroid, simplex lattice, and extreme vertices designs (2). Extreme vertices design uses a reduced number of required experimental trials to evaluate the observed responses which are then being utilized to predict the real responses. In this type of design a lower and upper values should be defined for each Mixture component (3). In this study the statistical method is based on extreme vertices design because it is concise and economical due to small number of required experiments. Thus, it is favorable in industrial manufacturing.

Povidone Iodine (PI) is the most common iodophore, releasing iodine slowly and is effective against bacteria, fungi, viruses, protozoa, cists and spores in pre operative site treatments, skin, periodontal and also in eye infections (4-7). Aqueous solutions of this agent are defined uncomfortable in transdermal application for wound disinfection, because of the low residence time on the skin and possible staining on the cloths (8).

The bactericidal effect of this agent is totally topical and transdermal absorption of the drug is unwanted and it has been previously reported that in some cases, its absorption into the systemic circulation leads to serious problems such as convulsion (9). Water removable ointment of PI introduced in USP may be a good choice in order to guarantee the topical action and acceptable residency along with easy removal through simple washing in some wide spread complications such as burning.

One of the most significant issues in pharmaceutical industries, prior to commercialization of a pharmaceutical preparation is the "preformulation" stage. To the best of our knowledge, far too little attention has been paid to verification of the software assisted statistical designs in preformulation studies. The main aim of this study was to report a step by step preformulation approach for a semisolid preparation based on a statistical mixture design and verifying the predictions made by the software with an *in-vitro *efficacy bioassay. Although a similar research has been done by Caffagi *et al*. on a lipophilic pharmaceutical compound (10), this type of study is unique due to verification of the predictions in practice followed by a bioassay and fulfills the requirements for reliance on the proposed method for industrial manufacturing purposes.

In detail, preformulation studies for a semisolid formulation of PI as a model drug was performed by applying a 4 factors, 4 levels extreme vertices mixture statistical design using Minitab software. Different molecular weights of polyethylene glycol, a widely used semisolid excipient(11, 12), were selected and the effect of their molecular weight on the available iodine, rheological properties and the water absorption capacity of the prepared semisolid formulations of PI were evaluated as response values, statistically. Finally, the software proposed an "optimized formulation" that was prepared in the laboratory and conformity of the measured responses with predicted values were evaluated. Subsequently, the efficacy of this formulation was bio-assayed using microbial tests on common human pathogenic bacteria and the resulted MIC values were compared with commercial solutions and ointments. 

## Experimental

*Materials*

Povidone iodine (PI) was purchased from (Behvazan, Iran), and commercial ointments and solutions (Label Claim = 10% PI) were obtained from (MundiPharma, Azerbayjan) and (Behvazan, Iran) respectively. All different PEG grades, Sodium thiosulphate (Na2S2O3.5H2O), sodium bicarbonate and sodium chloride, were prepared from (Merck-Germany).

Phosphate salts were obtained from (Scharlauchemie-EU). The water was deionized sterile water. Bacterial Species (ATCC 10536, ATCC 9027, ATCC 6538 and ATCC 10231) were all received from Iran scientific and industrial research center. 

*Methods*

*pH selection*

The effect of pH on the available iodine content in PI solutions was checked in order to provide a meaningful discussion on prepared formulations pH values.

Different pH values (2-7) according to USP pH range (12) were prepared and the available iodine was calculated accordingly. The pH values of all prepared formulations were recorded as a 5% aqueous solution (12) immediately after preparation and incubation at ICH accelerated condition.

*Design of experiment*

Extreme vertices mixture design (4 factors, 4 levels ) was applied for preformulation of a semisolid PI preparation using Minitab software and several formulations were prepared by different grades of Polyethylene glycols (liquid (PEG 400) and solid (1000, 2000 and 4000)) in order to prepare a water soluble ointment. The prepared formulations according to Minitab statistical mixture design were listed in Table 1. The variables were defined as different PEG contents in each formulation. Upper and lower limits were also defined for each component based on preliminary experimental results. The amount of PI, added water and NaHCO_3_ as a buffering agent was maintained constraint during formulation design.

**Table 1:** Composition of each formulation by name and percentage of each component proposed by statistical software design

**PEG 4000**	**PEG 2000**	**PEG 1000**	**PEG 400**	
60	15	3	7	F1
70	5	3	7	F2
66	5	7	7	F3
60	11	7	7	F4
70	5	7	3	F5
60	15	7	3	F6
64	15	3	3	F7
70	9	3	3	F8
65	10	5	5	F9
62.5	12.5	4	6	F10
67.5	7.5	4	6	F11
65.5	7.5	6	6	F12
62.5	10.5	6	6	F13
67.5	7.5	6	4	F14
62.5	12.5	6	4	F15
64.5	12.5	4	4	F16
67.5	9.5	4	4	F17

*Measurement of Responses*

*Available iodine*

The available iodine was assessed by USP method under PImonographusing sodium thiosulphate and starch as the titrant and the indictor solutions respectively (18).

*Rheological characteristics*

Viscosity measurements were performed using a cone and plate viscometer (HAAK, Germany). Temperature was maintained at 25°C and various shear rate moduluses or n (16, 22.5, 32, 45.2, 64, 90.5, 128, 181, 256, 363 and 512) were applied to test samples (prepared and commercial formulations) and stress coefficients or "s" were read throughout the instrument panel. Viscosity sensor system was named after manufacturer as pk1 and its physical characteristics along with coefficients in conversions have been listed in Table 2.

According to manufacturer manual, the shear rate (sec^-1^) can be computed by multiplying a constant sensor factor named "M" by previously mentioned shear rate modulus. Shear stress (Dyne/cm^2^) is similarly calculated by multiplying a constant sensor factor named "A" by "s". Finally, shear rates versus shear stresses, were plotted for all formulations. All Data were fitted to power law (14). Subsequently, the power (n) was calculated and recorded in Table 4.

Power law

F = K (R)^ n^

LnF = lnK + nLnR

In power low "K" is the flow consistency index (Pa.s^n^) or yield point and F and R are shear stress and shear rate respectively. Flow behavior index (n) is dimensionless.

The exclusive type of the rheological data forced us to employ difference factor formula to compare different formulations (15).

ƒ_1_= {[∑ _t=1_^n^ |R_t_-T_t_|] / [∑ _t=1_^n ^R_t_]} x100

In which R_t_ and are the viscosity of the reference and test preparation respectively and n is the number of data point.

**Table 2:** Physical characteristics and coefficients of Viscosity sensor system (pk1).

**Sensor system**	**Pk1**
Cone Radius (mm) Angle (^o^)	14 0.3
Plate Radius(mm)	14.5
Sample volume (cm^3^)	0.1
Temperature max (^o^C) Min (^o^C)	100 0
Sensor factors A (Dyne/cm^2^.sec.scale grad.) M (Min/sec) G (cp/Scale grad.*Min.)	810 20.6 3.940

*Water absorption measurements*

**Figure 1:**
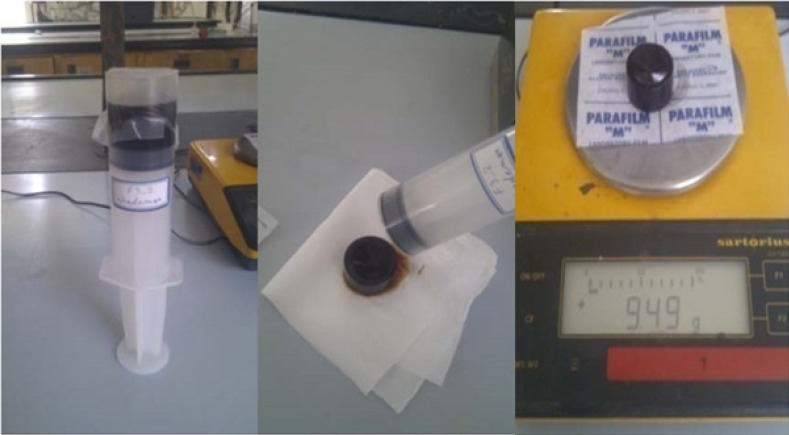
Demonstration of the water absorption content method according to Noda *et al*.

British Standard Institute Test Methods were applied to measure the amount of water absorbed to different prepared formulations (16). The results have been presented in table 1. Briefly medical grade syringes (50 cc volume) were filled with 2%w/v Agar in PBS and after gelling (about 20 mins), 1.5 gram of each semisolid formulation was carefully weighed and transferred onto the gel, inside the syringe. The opening of the syringe was fully covered using parafilm nylon and all syringes wee incubated at 25°C for 24 h (Figure 1). The amount of water absorbed to the gel was calculated gravimetrically by removing the semisolid preparation and weighing each gel individually (n = 3). Agar gel weight decrease was reported as the percentage of water absorbed per gram weight of the formulation ± SD. 

*Statistical analysis*

Software designs a set of experiments in a small region of real conditions. As mentioned before all formulations were prepared and 4 different responses including; available iodine, viscosity (N index and yield value) and water absorption capacity were measured and analyzed in order to prepare a prediction chance for the software to propose the "Optimized formulation". The prediction was combined with stability data. All response values were also recorded after 30 and or 60 days incubation at room and accelerated conditions (40°C) according to ICH guidelines. In this section the software fits the acquired responses to different regression models (mixture regression, stepwise, forward and backward) and best fit is selected according to RSQ and MPE values. The basic equations and detailed information have been mentioned by Cafaggi *et al*. (10). After successful model development, the plots were used to compare each component effect on the responses. Finally, an optimized formulation was proposed by the software using design of experiment approach. The proposed optimized formulation was prepared in the laboratory and the real responses were calculated and compared with software predictions.

*Water absorption rate*

**Figure 2:**
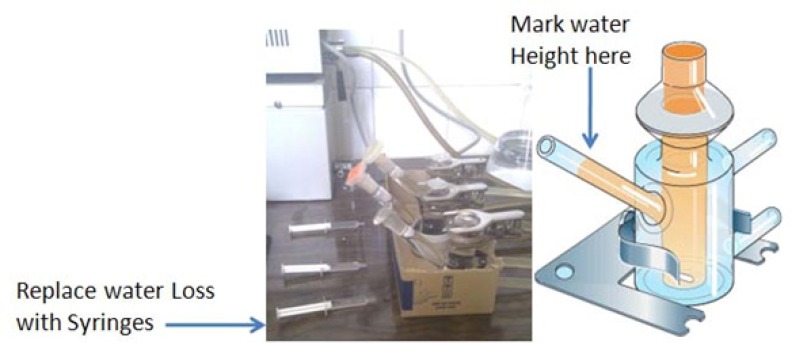
Demonstration of Water absorption rate apparatus according to Noda *et al*.

Beside the water absorption capacity which was measured for all formulations, water absorption rate was also calculated according to Noda *et al* only for the optimized formulations in order to compare them with commercial available preparations (16). Briefly the prepared formulation was transferred on a micro dialysis membrane which was previously mounted on a diffusion Franz cell filled with PBS. According to Figure 2, the height of the solvent in the pipe type port of the cell was marked and the reduced solvent which was absorbed to the ointment was replaced by a pre weighed syringe filled with PBS, in predetermined time intervals. The syringe weight loss was recorded and cumulative amount of absorbed water normalized to surface area (mg/cm^2^) was plotted against square root of time according to Kawashima *et al*. (17) and the slope was calculated as the water absorption rate (mg/cm^2^/Min^0.5^)and recorded for prepared and commercial formulations.

*Efficacy measurements*

The microbial tests were employed to check the efficacy of the optimized formulation along with commercial ones, using MIC values against Escherichia coli (ATCC 10536), pseudomonaaeruginosa (ATCC 9027), staphylococcus aureus (ATCC 6538) and Candida albicans (ATCC 10231). Stability sample was also tested for efficacy and comparisons were made. Microbial inoculums were prepared as 1.5 * 10^8^ CFU/mL which was equivalent to 0.5 McFarland of standard. All samples (semisolids and solution) were prepared as a 10 % aqueous solution. 

Serial dilution method in Nutrient broth was employed for MIC calculation. MBC (Minimum bactericidal concentration) was measured using agar media.

## Results and Discussion

*pH selection and measurements*

**Figure 3:**
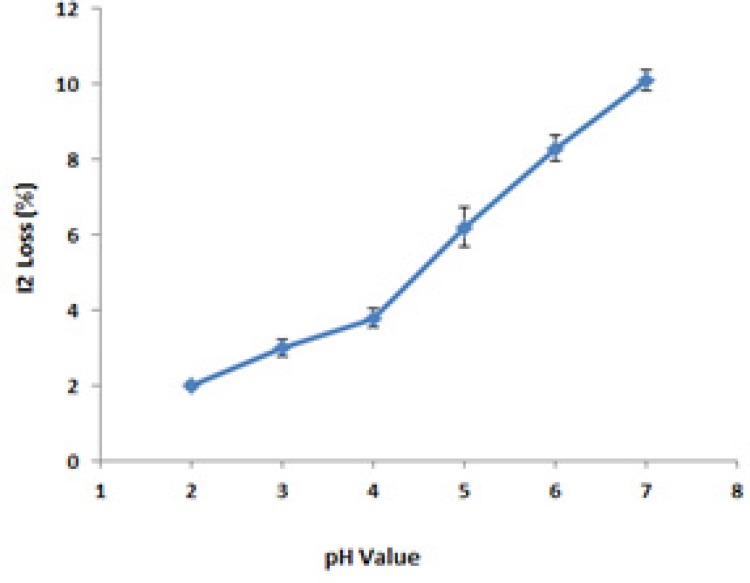
The effect of pH on available iodine

USP provides a wide acceptable pH range for PI ointment (pH = 1.5-6.5). As shown in Figure 3, the best pH value was concluded to be equal to 4. All formulations were prepared according to this finding.

Table 3 lists the mean pH values (n = 3), for all formulations (F1-F17) immediately after production and also after 30 and 60 days incubation at 40°C, 75 % RH. According to results, a slight decrease ranged from 0.01 to 0.25 in pH unit was observed.

Povidone Iodine is an Iodophore that consists of Poly vinyl pyrolidone along with (I_2_) or (HI_3_). The germicidal activity of this Iodophore depends on the free iodine concentration (Available Iodine) in the solution. This can explain the effect of pH on the PI activity. A slight decrease in the pH value of the prepared formulations can be explained by the fact that, in aqueous solutions Iodine can exist in many different forms, but the reactions that may lead to pH decrease can be shown as follow:


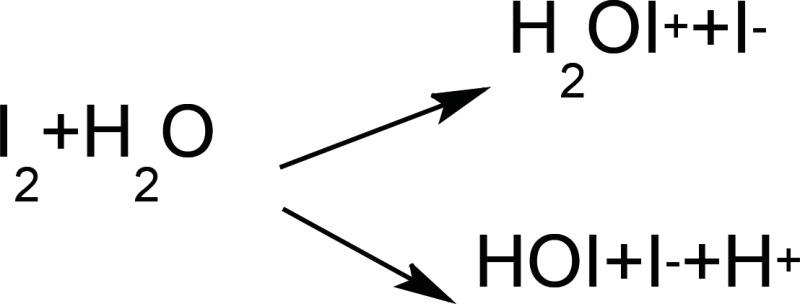


It should be kept in mind that, I_2_, I^-^ and HOI exhibit the germicidal activity (20). Although a slight pH decrease was observed, all formulation did have the acceptable pH according to previously mentioned criteria.

**Table 3:** Mean pH values for all formulations (F1-F17) immediately after production and also after 30 and 60 days incubation at 40°C, 75% RH.

	**pH(Mean ± SD)**		
**Formulation**	**0 Day**	**30 Day**	**60 Day**
F1	3.60±0.01	3.49±0.01	3.64±0.01
F2	3.54±0.01	3.31±0.01	3.45±0.01
F3	3.50±0.01	3.31±0.01	3.42±0.01
F4	3.54±0.01	3.30±0.01	3.40±0.01
F5	3.54±0.01	3.40±0.01	3.51±0.01
F6	3.52±0.01	3.45±0.01	3.60±0.01
F7	3.60±0.01	3.54±0.02	3.64±0.01
F8	3.60±0.01	3.54±0.01	3.62±0.02
F9	3.60±0.01	3.54±0.01	3.58±0.01
F10	3.60±0.03	3.51±0.01	3.57±0.01
F11	3.61±0.02	3.55±0.01	3.59±0.01
F12	3.63±0.01	3.56±0.01	3.60±0.01
F13	3.65±0.02	3.57±0.01	3.58±0.00
F14	3.69±0.02	3.56±0.01	3.58±0.00
F15	3.65±0.02	3.54±0.01	3.59±0.01
F16	3.66±0.02	3.56±0.01	3.61±0.01
F17	3.67±0.02	3.58±0.01	3.61±0.01
Fx	3.99±0.06	3.98±0.01	3.99±0.02

In order to explain the pH variations, each PEG aqueous solution (5%w/v) was individually tested and the resulted pH values for PEG 400, 1000, 2000 and 4000 were 5.1, 4.1, 5.5 and 5.9 respectively. The pH value for different grades and different manufacturer of PEG may be different, due to the variations in manufacturing processes (pH = 4-7).

*Measurement of Responses*

*Available iodine*

**Figure 4:**
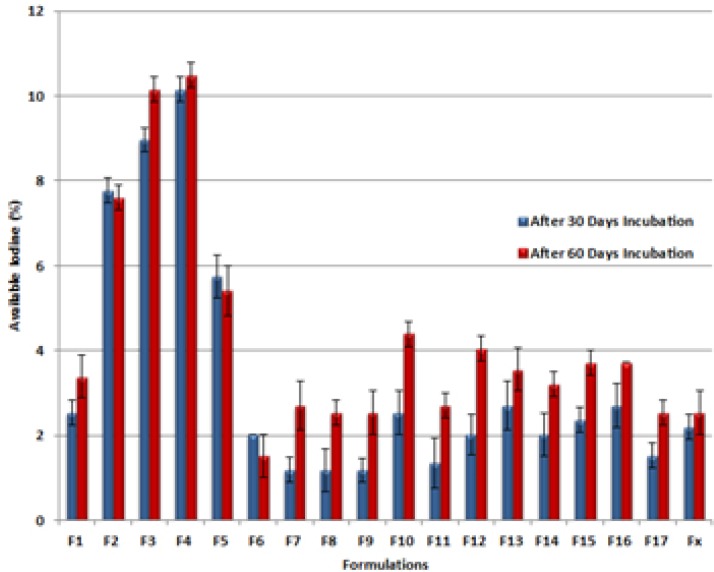
Differences of available iodine content in prepared formulations (F1-F17) and commercial one (FX) after 30 and 60 days incubation at 40°C, 75 % RH with the initial value

As mentioned before, all prepared formulations (F1-F17) were subjected to Available iodine measurement immediately after preparation and 30 and 60 days after incubation at accelerated ICH condition (40°C, 75 % RH). Figure 4 illustrates the differences of remained available iodine content in prepared formulations (F1-F17) and a commercial one (FX) after 30 and 60 days incubation at 40°C, 75% RH. All experiments were done at least 3 times (n = 3).

Available iodine content of the preparations immediately after production varied in the range of 98.98-103.72 % of the labeled claim. Although the commercial PI ointment was within the labeled shelf life but the available iodine was about 89.5%. After incubation for 30 and 60 days, the range was changed to 93.57-99.83 and 93.23-98.47 respectively. According to USP the acceptable assay results lies between 85 to 120% and it can be concluded that all formulations were accepted even after incubation in ICH accelerated stability condition. The presence of "Significant change" was also checked (12, 13) and the results indicated that only a few formulations had available iodine below 95 percent and thus were not stable (F2, F3, F4 and F5). 

This instability may be explained by possible physical or chemical linkage of Iodine to high molecular weight PEGs as all the instable formulations had higher contents of this component. Reducing agents such as impurities arising from polymerization process and known as by products have an adverse effect on the stability of the Iodine by incorporating in Red/Ox reactions (19). Due to more sever polymerization needed in high molecular weight PEGs this can be another reason to explain the results. Another explanation can be made by micro environmental pH variations in the presence of high molecular weight PEGs that has a great effect on the amount of free iodine content (20).

*Rheological properties*

The pesudoplastic behavior was revealed for all prepared formulations and the commercial ointment. The difference between measurements was considered statistically significant indicating that selected time points are able to differentiate the formulations and finally based on the rheological behavior the similar formulations to commercial ointment were identified. 

**Figure 5:**
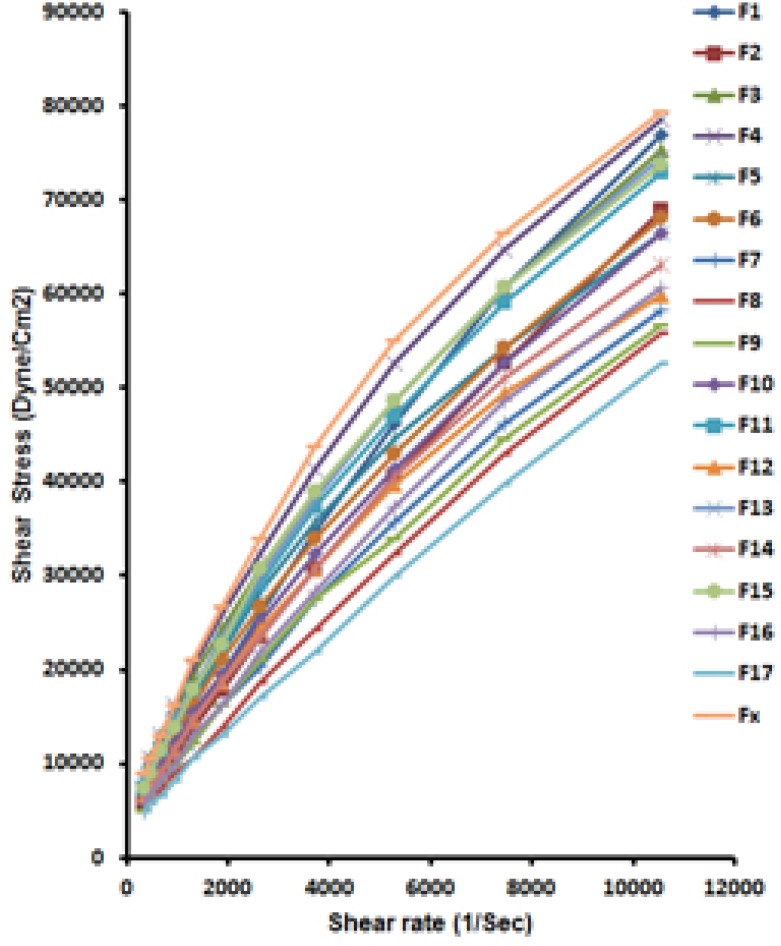
Shear rate versus shear stress for all formulations (F1-F17).

Figure 5 illustrates; shear rates versus shear stresses for all formulations along with the commercial one. Table 4 shows the results of fitting data to power low listing flow behavior index (n) and yield point (M) for prepared formulations along with a commercial one.

Figure 5 and Table 4 both indicate a Pseudoplastic flow with a yield value (n or Flow behavior index < 1).

**Table 4.** Flow behavior index (n) and yield point (M) for all formulations and a commercial preparation

	**Formula**	**n**	**M(Dyne/Cm** ^2^ **)**
F1	Y = 86.394x ^0.7189^	0.7189	86.394
F2	Y = 117.88x ^0.6728^	0.6728	117.88
F3	Y = 230.16x ^0.6114^	0.6114	230.16
F4	Y = 186.63x ^0.6429^	0.6429	186.63
F5	Y = 69.532x ^0.7206^	0.7206	69.532
F6	Y = 74.73x ^0.7205^	0.7205	74.73
F7	Y = 48.688x ^0.7565^	0.7565	48.688
F8	Y = 43.609x ^0.7587^	0.7587	43.609
F9	Y = 74.73x ^0.7205^	0.7205	74.73
F10	Y = 59.459x ^0.747^	0.747	59.459
F11	Y = 79.05x ^0.7108^	0.7108	79.05
F12	Y = 95.926x ^0.7108^	0.7108	95.926
F13	Y = 105.26x ^0.6997^	0.6997	105.26
F14	Y = 74.959x ^0.7203^	0.7203	74.959
F15	Y = 67.214x ^0.7349^	0.7349	67.214
F16	Y = 42.104x ^0.7793^	0.7793	42.104
F17	Y = 97.513x ^0.6779^	0.6779	97.513
Fx	Y = 143.98x^0.6781^	0.6781	143.98

Rheological properties of each formulation immediately after preparation and then after incubation for 30 and 60 days (data not shown) were statistically significant (p < 0.05) based on ANOVA. Difference factors shown in table 5 compare different formulations with an innovator commercial preparation.

**Table 5:** Difference factor for all formulations compared with a commercial preparation.

	**Difference Factor**
F1 & Fx	15.27
F2 & Fx	21.00
F3 & Fx	8.59
F4 & Fx	4.30
F5 & Fx	30.67
F6 & Fx	25.78
F7 & Fx	34.37
F8 & Fx	39.86
F9 & Fx	25.78
F10 & Fx	26.49
F11 & Fx	27.45
F12 & Fx	11.93
F13 & Fx	11.69
F14 & Fx	25.78
F15 & Fx	24.58
F16 & Fx	31.98
F17 & Fx	31.50

When a Difference factor is less than 15% two compared formulations can be estimated as similar (21). According to table 5 similar formulations in the case of rheological properties were F3, F4, F12 and F13. More detailed discussions have been provided in previous sections. 

*Water absorption measurements*

**Figure 6:**
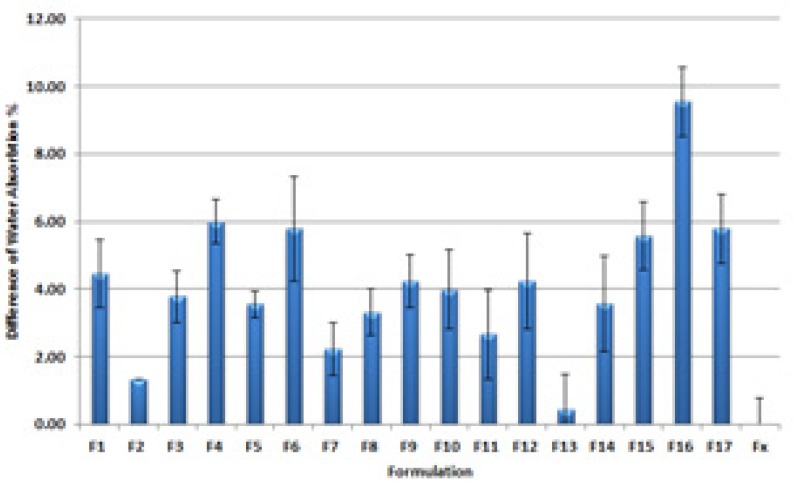
Differences between Water absorption contents after 30 with 60 days incubation for all prepared formulations and a commercial ointment

Figure 6 shows the differences between Water absorption contents of all prepared formulations and a commercial ointment.

Water absorption percentage (WA%) is a key factor in the efficacy of the prepared PI ointment in exudative wound disinfection. Water absorbing ointments manage the exudates more efficiently as they remove the aqueous exudate form wound nearby (22). Recently Noda *et al*. presented a 26 ± 0.2% and 76 ± 0.5% water absorption capacity for different iodophoreointments (16). Our prepared formulations had a WA% ranged from 14.66 to 53.55 percent. F16 and F13 had respectively the maximum and minimum WA% in tested formulations.

F16 with high WA% contains medium content of PEG 400 and PEG 1000 along with a high content of PEG 2000 and PEG 4000. This can be explained by the fact that higher polymerization in solid PEGs lead to higher water absorption in the final formulation. But there is no simple conclusion when all the mixtures where designed with higher solid components. Thus it is better to mention the best composition of PEG400: PEG 1000: PEG 2000: PEG4000 to be 4: 4: 12.5: 64.5.

*Analysis of Mixture design*

*Model fitting*

The best fit was selected by applying different models in a linear or quadratic term and the results are shown in table 6.

**Table 6:** Best fit results for each response value

**P-Value**	**MPE%**	**R-Square**	**Model Fitting**	**Term**	**Time**	**Test**
0.032	0.94	48.01%	Mixture Regression	Linear	60 Days	Assay
0.035	0.84	90.42%	Backward	Linear	30 Days	N Index
0.013	6.13	93.19%	Forward	Linear	30 Days	Yield Point
0.012	7.10	71.00%	Backward	Linear	60 Day	Water Absorption

*Plots*

All useful definitions of different plots have been presented elsewhere (23). Briefly Response Trace Plot (RTP) shows each component effect on the response value. In this research four different response values were evaluated and each mixture had contained four components which were designed statistically using Mixture design approach. All plots perform the prediction based on the selected model in model fitting process. Data interpretation is described in details in Minitab software help (24).

*Response Trace Plots*

**Figure 7:**
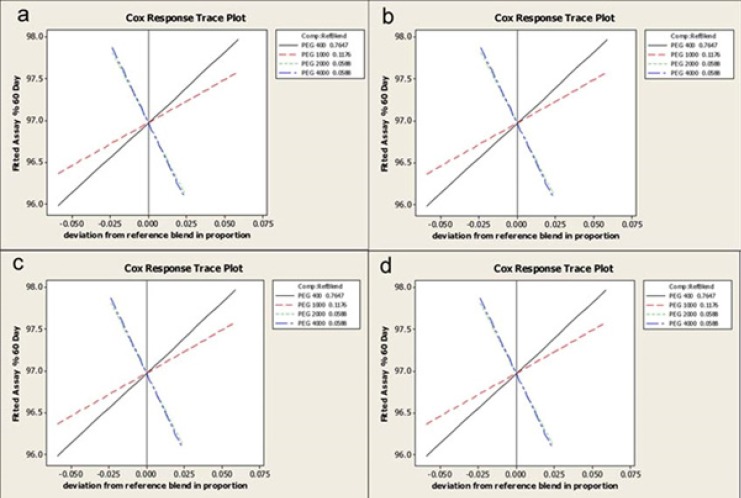
Response trace plots depicting the effect of each component on the evaluated responses a) available Iodine, b) yield value (M), c) n index, and d) water absorption content

The effects of each component on the different response values; total available iodine, rheological properties and water absorption, have been shown in Figure7.

*Interpretations*

PEG 400 has the main effect on Available iodine response due to its high slope value and accordingly the effect of PEG 2000 and 4000 are minimum and also similar to each other. Negative slope for PEG 4000 and 2000 traces, indicates an inverse proportion of these components on available iodine response.

The effect of PEG 1000 on N index as another response value shows a plateau (Figure 7). This means that by increasing PEG 1000 the N index increases and finally reaches to a plateau and after that increasing the component content leads to the decrease in the response value. Negative slopes can be seen for PEG 4000 and 2000 traces.

According to findings when the amount of PEG 400 decreases and all other components increases, the yield value (m) increases.

Water absorption of the prepared semisolid formulations increased by increasing the amounts of solid components.

Although conclusions made from these plots are so useful but the software uses these plots only to introduce a schematic representation of its predictions.

*Overlaid Contour Plots*

**Figure 8:**
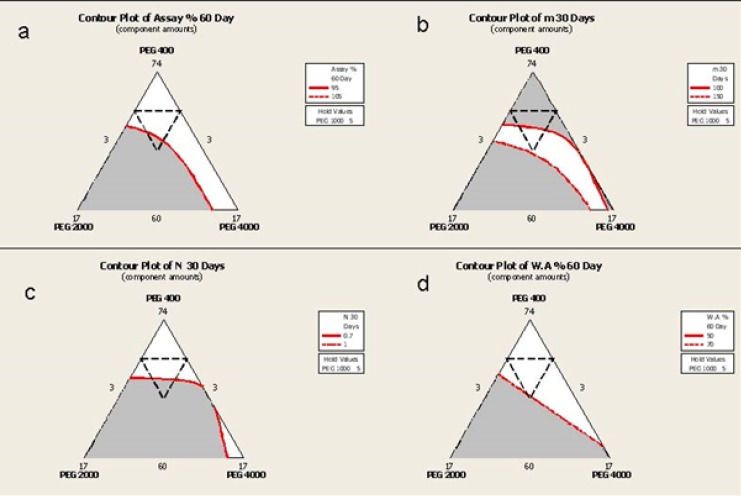
Overlaid counter plots depicting the effect of each component on the evaluated responses a) available Iodine, b) n index, c) yield value (M) and d) water absorption content

Overlaid Counter Plots (OCP) are three dimensional representations of the effects of each component on a response value for a maximum of three components. OCPs have been shown in Figure 8. This is a topographic illustration of the results and totally adds the color to the graph for better explanation. The internal dotted line triangle shows the region that is prepared in the laboratory and the remaining parts have been concluded by software prediction. The red lines inside is defined by the researcher as the desired values.

 In available iodine the defined desired value is based on the definition of "Significant Change" according to ICH stability Guidelines (95% assay). 

From a practical point of view, the appropriate yield value in delivering the semisolid preparation through its container has been ranged from 100-150 which allows the consumer to use the product by applying a minimum pressure to the container. In this research this is defined as the desired value for "m".

N index near to unit depicts a Newtonian behavior but as the prepared semisolids do not completely comply with this law and are categorized as Pseudoplastic systems, a desired value between 0.7-1 was defined. 

According to previously published researches, the best value for water absorption was between 50-70%. 

*Optimization*

After modeling the design and finding out the acceptable range of the evaluated components, the software proposes an optimized formulation (F_O_). This formulation with composite desirability equal to 1 was prepared in the laboratory and all physicochemical properties as well as the efficacy and waster absorption rate was calculated and recorded (Table 7).

**Figure 9:**
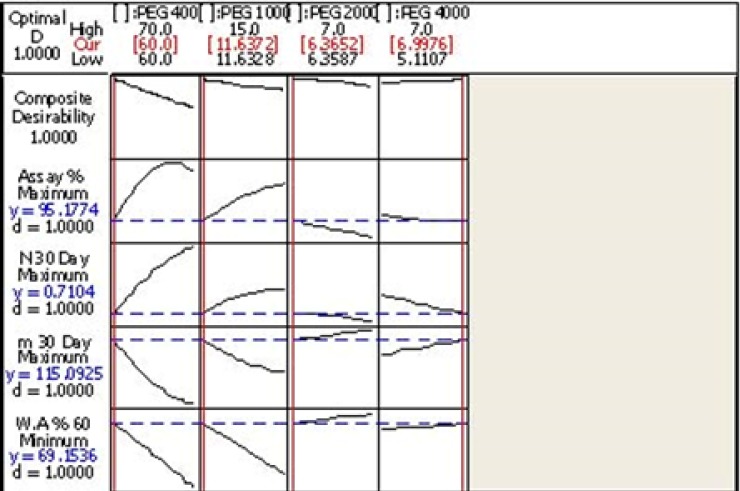
Optimization Results

**Table 7:** The physicochemical properties of F_0_ immediately after preparation and one year after incubation at ICH accelerated condition

**365 Days**	**0 Day**	**Test**
103.18± 0.28	106.08 ± 0.32	Available Iodine
3.33 ± 0.01	3.32 ± 0.01	pH
0.6956	0.6952	N
157.97	153.93	M
70.12 ± 0.43	68.68 ± 0.67	W.A capacity

*Water absorption rate*

**Figure 10:**
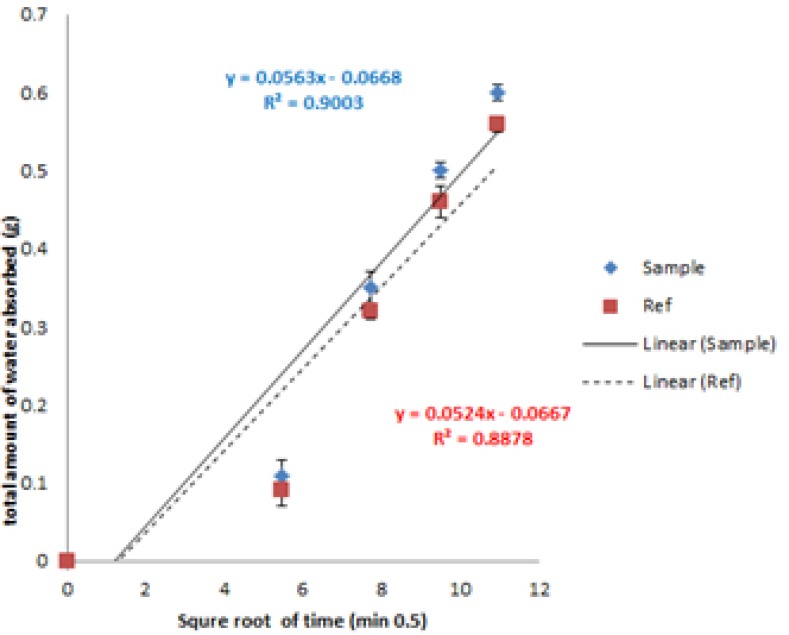
Water absorption rate for Fo (diamond) and commercial formulation (Square).

Water absorption rate was performed in order to compare optimized formulation with commercial available preparations (16). Cumulative amount of absorbed water normalized to surface area (mg/cm^2^) was plotted against square root of time in Figure 10. The calculated slope was normalized to surface area (6.1544 cm^2^) and the rate was reported as 9.14 and 8.51 (mg/cm2/Min0.5) for prepared and commercial formulations, respectively.

*Efficacy measurements*

Different researchers have previously examined the efficacy of the novel transdermal dosage forms to ensure the desired therapeutic outcomes (25).

**Figure 11:**
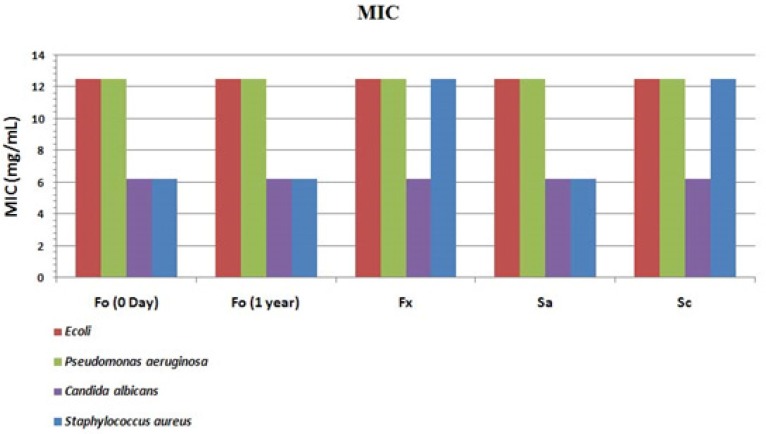
MIC comparison for optimized formulation (Fo) immediately after preparation (0day) and one year incubation (1 year), Commercial Mondipharma ointment (Fx), pH adjusted aqueous solution (Sa) and Commercial Behavazan aqueous preparation (Sc).

MIC values were calculated to compare the efficacy of the optimized formulation with commercial ones (aqueous or semisolid) and also controls (Figure 12). The lower the MIC values indicate the fewer drugsarerequired to inhibit the bacterial growth in harvesting media. Results indicate a similar antiseptic effect of optimized formulation (Fo) compared to a commercial preparation (Fx) but a more potent action on staphylococcus aureus. This provides a very important advantage because this bacterium belongs to skin normal flora and is responsible for damaged skin infections in some complications such as; burning, trauma or operative site infections and thus its suppression is needed in dermal wound healing processes. The accelerated stability study of Fo revealed no significant change according to ICH guidelines. No standard deviation of the results is presented because in each replicated sample (n = 3) the inhibitory concentration, was exactly the same. For better explanation the results for a pH adjusted aqueous solution (Sa) prepared in the laboratory and a commercial Behavazan aqueous preparation (Sc) are also presented in Figure 11. The variations in the efficacy of commercial preparations may be due to inappropriate pH value, which leads to the iodine inactivity. Theseresultsareconsistent withthe previous results gained for the available iodine. This phenomenon is fully discussed in "pH selection and measurements" section of this report. 

## Conclusion

This was the first research reporting a step by step preformulation approach for a semisolid preparation based on a statistical software design and verifying the predictions made by the software using an *in-vitro *efficacy bioassay.

As shown, the extreme vertices mixture design needs the operator consciousness in selecting the appropriate model and in defining the desired response values. Without this human logical interfere the software may not be successful in providing a good prediction.

The results showed a suitable comply between the design and practice. This may lead to use more logical steps in industrial pharmaceutical designs and saving time and also the most important factor Money! 
